# Prognostic Value of Serum Total Bilirubin after Percutaneous Coronary Intervention in Patients with Acute Coronary Syndrome

**DOI:** 10.1155/2019/5243589

**Published:** 2019-05-28

**Authors:** Chengchun Tang, Hao Qian, Dong Wang, Yong Qiao, Gaoliang Yan

**Affiliations:** ^1^Department of Cardiology, Zhongda Hospital, Nanjing, China; ^2^Southeast University Medical School, Nanjing, China

## Abstract

**Backgrounds:**

Previous studies have reported a relationship between serum total bilirubin (STB) and coronary artery disease (CAD). However, the relationship between STB and prognosis of patients with acute coronary syndrome (ACS) after percutaneous coronary intervention (PCI) remains inconclusive. The present study aimed to evaluate the relationship between STB level and prognosis of PCI in patients with ACS.

**Methods:**

In total, 2850 ACS patients who underwent PCI at the Affiliated Zhongda Hospital of Southeast University from June 2009 to Jan 2017 were included in the study. Twenty-four-hour STB, 30-day, and 1-year postoperative major adverse cardiovascular events (MACE) were recorded. Subjects were assigned to one of three groups based on STB: Group A (STB ≤ 9.6 *μ*mol/L), Group B (9.7 *μ*mol/L < STB ≤ 15.4 *μ*mol/L), and Group C (STB ≥ 15.5 *μ*mol/L). COX survival analysis was subsequently used to investigate the relationship between the incidence of MACE and STB in the three groups.

**Results:**

A total of 2770 subjects were successfully followed up; within 1 year after PCI, 115 (4.15%) subjects died and 191 (6.90%) subjects experienced MACE. One-year follow-up results showed that the incidence of MACE decreased significantly as STB increased; the risk of Group A was 2.002 times that of Group C (95% CI: 1.342-2.986). Cardiac mortality also decreased with increasing STB; the risk of Group A was 3.403 times that of Group C (95% CI: 1.319-8.785).

**Conclusion:**

Lower mortality and MACE incidence rates were found in patients with higher STB within 1 year. Therefore, STB is highly recommended as an independent long-term prognosis predictor of PCI in patients with ACS.

## 1. Introduction

Acute coronary syndrome (ACS) refers to a spectrum of clinical presentations caused by acute myocardial ischemia [1]. It is believed that the rupture or erosion of unstable atherosclerotic plaques is the main pathological basis of the incidence of ACS. At present, percutaneous coronary intervention (PCI) is the principal revascularization strategy employed in the treatment of ACS. The prognosis of PCI in patients with ACS is a key clinical issue, and some studies have indicated that C-reactive protein (CRP), troponin (Tn), and D-dimers are associated with the prognosis [2]; however, a gold standard has not been established.

A large number of domestic and foreign studies have found that STB has antioxidant, free-radical scavenging [3, 4], and antiatherosclerosis effects [5] and is negatively correlated with the incidence and prognosis of coronary atherosclerotic heart disease [6]. However, few studies have investigated the relationship between STB and prognosis of PCI in patients with ACS, and a certain degree of controversy exists in the reported results [7–9]. Therefore, the present study aimed to investigate the relationship between STB and the prognosis of PCI in patients with ACS.

## 2. Materials and Methods

### 2.1. Study Population

This study is a retrospective study of 2,850 consecutive patients diagnosed of ACS from June 2009 to Jan 2017 in Zhongda Hospital. The inclusion criteria are as follows: (1) diagnosed as ACS according to the World Health Organization standards; (2) PCI was performed after admission. The exclusion criteria are as follows: (1) life expectancy is less than 1 year due to diseases such as malignant tumors; (2) have liver and gallbladder diseases such as hepatitis and gallstones; and (3) subjects with serum STB > 34.2 *μ*mol/L (2 mg/dl) were excluded due to possible chronic liver disease or Gilbert's syndrome [10].

### 2.2. Baseline Information and Laboratory Examinations

For all subjects, general clinical data and laboratory and imaging results were entered; clinical data were from the hospital medical record system. All blood tests were performed at the Department of Clinical Laboratory, Affiliated Zhongda Hospital of Southeast University. All patients underwent color Doppler echocardiography (performed at the Color Ultrasound Room, Department of Cardiology, Zhongda Hospital) within 72 hours of admission, and the left ventricular ejection fractions (LVEFs) were recorded.

### 2.3. Follow-Up and Study Endpoints

Of 2,850 patients, 2,770 ones completed 1 year of follow-up. Follow-up was conducted through outpatient visits or by phone. The primary endpoint was cardiac death and the secondary endpoint was other MACE; for each event, the time of event occurrence was recorded. MACE include cardiac death, myocardial infarction, revascularization, and stent thrombosis.

### 2.4. Data Analysis

The 2770 subjects were assigned to one of three groups according to the STB range: Group A: 923 subjects (STB ≤ 9.6 *μ*mol/L), Group B: 924 subjects (9.7 *μ*mol/L < STB ≤ 15.4 *μ*mol/L), and Group C: 923 subjects (STB ≥ 15.5 *μ*mol/L). Data analysis was performed using SAS (9.4, North Carolina State University). For quantitative data that followed a normal distribution, comparison between multiple groups was performed by analysis of variance; for data that followed a nonnormal distribution, comparison between multiple groups was performed by rank comparison tests. For categorical variables, the chi-square test was used to compare the composition ratios among the groups. Multivariate analysis and survival analysis were, respectively, performed using the COX proportional hazards model for those indicators that are meaningful after univariate analysis. Statistical significance was defined as p < 0.05.

## 3. Results

The 2770 subjects were divided into three equal groups. Group A included 923 patients (mean age, 60 ± 11.47 years; males, 69.88%), Group B included 924 patients (mean age, 60 ± 11.32 years; males, 69.05%), and Group C had 923 patients (mean age, 60 ± 11.48 years; males, 70.64%). The baseline data in Table 1 shows that there were no statistical differences in age, sex, and BMI among the three groups, whereas the differences in other variables such as diabetes, renal insufficiency, troponin, HBA1C, STB, serum direct bilirubin (SDB), ALT, aspirin, and statins were statistically significant.

As shown in Tables 2 and 3, there was no significant difference in MACE incidence and cardiac mortality between the three groups within 30 days. The results of one-year follow-up had also been shown in Tables 2 and 3; as we can see, the incidence of MACE in patients with lower STB was significantly higher than those in patients with higher STB; the risk of Group A was 2.002 times that of Group C (95% CI: 1.342-2.986). At the same time, the risk of Group A was 3.403 times that of Group C (95% CI: 1.319-8.785) regarding cardiac death.

Tn, AST, and LVEF were found to be independent predictors of death within 1 year in patients, with Tn positively correlated and LVEF and AST negatively correlated to the risk of cardiac death.

Table 4 presents an analysis of the difference in STB level among patients with different diseases. The results show that only diabetes had an effect on the STB levels of the ACS patients. The STB levels of the ACS patients without diabetes were higher than those of the ACS patients with diabetes (p=0.028).

## 4. Discussion

The present study found that STB was not significantly related to the occurrence of MACE and cardiac death within 30 days but was significantly negatively correlated within 1 year. Therefore, we believe that STB can be used as an independent predictor of long-term prognosis after PCI in ACS patients.

At present, the mainstream view of atherosclerosis is that it is mainly associated with an inflammatory injury of the vascular endothelial cells [10–13]. Several studies have asserted that the possible reason for which the incidence and mortality of coronary heart disease are correlated with total bilirubin concentration is that a reduced serum STB indicates the presence of an inflammatory response [14]. Heme oxygenase (HO) is the rate-limiting enzyme in the series of reactions and plays the essential role in the formation of bilirubin. Of two isoforms of HO, elevated expression levels of the HO-1 in response to several stimuli provide cardioprotection by anti-inflammatory, antiapoptotic, antiproliferative, and antithrombotic effect [15]. The low levels of bilirubin may partially mirror an impaired HO-1 response to vascular injury, resulting in a reduced antioxidant and anti-inflammatory ability of body, which links lower STB and higher risk of long-term mortality in coronary artery disease (CAD) patients [10]. The results of a study by Gullu et al. indicated that bilirubin may indirectly improve coronary microvascular dysfunction and impaired coronary flow reserve through its anti-inflammatory properties, thereby reducing the incidence and development of coronary heart disease [16].

In ACS patients, the coronary arteries are severely narrowed or even completely blocked. After the blood vessels have been opened by PCI, reperfusion injury may occur when blood flow is restored to ischemic cardiac cells. The peroxidation effects caused by free radicals have been regarded as key mechanisms of reperfusion injury [17]. Oxidative stress is a stress-induced chronic inflammatory response that arises in cells after vascular endothelial injury. The reactive oxygen species (ROS) generated in cells through oxidative stress in cells play an important role in the oxidative stress response. Low levels of ROS in the body can act as signaling molecules, while large amounts of ROS may have adverse effects on cells [18]. The binding of bilirubin and albumin produces an asymmetric complex, which gives rise to active hydrogen atoms in the molecular structure that can easily bind with oxygen free radicals, thereby producing a strong antioxidant effect. In addition, bilirubin can also inhibit the formation of oxidized low-density lipoprotein (ox-LDL) through its antioxidant effect [19].

Some studies have reported a significant acceleration of coronary artery restenosis, which commonly occurs 3-6 months after angioplasty in patients who underwent PCI. Smooth muscle cell proliferation and migration have been regarded as key mechanisms for the occurrence of post-PCI restenosis [6]. Öllinger et al. found that bilirubin can inhibit the proliferation of vascular smooth muscle cells, thereby reducing the extent of vascular stenosis [20]. A study by Kuwano et al. also indicated that higher concentrations of bilirubin can reduce the incidence of coronary in-stent restenosis through its inhibitory effects on vascular smooth muscle cell proliferation [21].

Besides the aforementioned mechanisms, we believe that the negative correlation between STB and prognosis in ACS patients shown by the present study may also be due to the endothelial function-enhancing effects of bilirubin. A study by Erdogan et al. indicated that patients with chronic total coronary occlusion who had higher STB within the normal range had better collateral development compared with patients with lower STB, which is suggestive of the endothelial function-enhancing effects of bilirubin [22]. In a study by Yoshino et al., it was also reported that patients with higher bilirubin concentrations may have better endothelial function, which provides resistance against the impairment of coronary artery endothelial function [23].

This study found that STB was inversely related to the patient's long-term prognosis, but this correlation was not linear. Compared with patients in the hyperbilirubin group, the incidence of MACE was significantly higher in the lower bilirubin group within 1 year, but not in the middle bilirubin group. In addition, the follow-up results within 30 days showed no statistical difference between the three groups, which may be due to the short-term stress response caused by the occurrence of ACS and surgery, which masked the protective effect of hyperbilirubin, so the difference between groups was not obvious in the short term.

There is still some controversy about the relationship between STB and the prognosis in ACS patients. Kaya et al. demonstrated that STB positively correlated with the burden of coronary atherosclerosis in both STEMI and non-STEMI so that elevated STB was independently associated with higher incidence of short-term mortality following AMI [24]. Sang-Ryul Chung et al. find that STB is a powerful prognostic marker; inclusion of this can improve prediction of in-hospital MACE in patients with STEMI undergoing primary PCI [25]. However, studies of Zhang et al. show that high STB concentration is associated with lower MACE in patients with ACS after PCI [26]. In another study, Yao et al. demonstrate that low STB before PCI is an independent predictor of long-term adverse clinical outcomes in patients with angina pectoris; however, no association was found with in-hospital mortality and myocardial infarction [27]. The discrepancies in the reported study outcomes could be due to the following reasons. Firstly, the number of cases studied could be inadequate and, thus, lack representativeness. Secondly, differences exist in study populations of different studies. The study population in the present study included ACS patients, while other studies focused on STEMI or NSTEMI patients; therefore, the obtained results may not be consistent. Thirdly, follow-up durations were not standardized across studies. Therefore, follow-up involving longer durations and more subjects is required to enhance the credibility of such studies.

The study also found that Tn, AST, and LVEF are always associated with patients with MACE and cardiac mortality. Among them, Tn is positively correlated: the higher the patient's Tn, the worse the prognosis, and Tn reflects the degree of myocardial damage in patients, which is consistent with the results of the study. LVEF reflects the patient's cardiac function. The higher the LVEF, the smaller the cardiac function damage and the better the prognosis. AST is also a marker of myocardial damage, mainly increases in the case of cardiac injury, and its relationship with the prognosis of patients remains to be further studied.

The present study had several limitations. Firstly, there was only single bilirubin data at admission; no repeated testing was done during long-term follow-up, which may have resulted in biases in the study results. Secondly, as this was a single-center clinical study, population limitations may exist in the cases studied, and the conclusions derived from the study may differ from certain multicenter studies. Thirdly, the follow-up period in this study was limited to 1 year, which is not entirely representative of the prognosis in the subjects. Therefore, further follow-up and investigation are required.

## 5. Conclusion

The results of this study indicated that MACE and cardiogenic mortality in the higher bilirubin group were significantly lower than those in the lower bilirubin group within 1 year. Therefore, STB is highly recommended as an independent predictor of long-term prognosis in ACS patients.

## Figures and Tables

**Table 1:** Demographic, clinical, and laboratory characteristics according to STB.

Variable	Group A	Group B	Group C	P*-*value
	(N= 923)	(N= 924)	(N= 923)	
Age, year	60 ± 11.47	60 ± 11.32	60 ± 11.48	0.514
Men, n (%)	645 (69.88 %)	638 (69.05 %)	652 (70.64 %)	0.757
BMI, kg/m^2^				0.323
Lower than normal, n (%)	315 (38.60 %)	318 (41.68 %)	283 (36.71 %)	
Normal, n (%)	434 (53.19 %)	386 (50.59 %)	430 (55.77 %)	
Higher than normal, n (%)	67 (8.21 %)	59 (7.73%)	58 (7.52 %)	
*Clinical variables*				
Hypertension, n (%)	585 (63.38 %)	561 (60.71 %)	551 (59.70 %)	0.245
Diabetes, n (%)	177 (19.22 %)	126 (13.76 %)	144 (15.67 %)	0.006
Smoking, n (%)	418 (46.24 %)	446 (49.06 %)	433 (47.53 %)	0.483
Hyperlipidemia, n (%)	322 (34.92 %)	302 (32.68 %)	305 (33.08 %)	0.554
Renal insufficiency, n (%)	508 (55.52%)	448 (48.85%)	491 (53.60%)	0.013
LVEF, n (%)	50. 89 ± 11.13	50. 88 ± 11.20	50.75 ± 10.92	0.954
*Laboratory values*				
Troponin, ng/mL	0.40(0.03, 3.6)	1.20(0.31,4.70)	0.98 (0.03,4.60)	0.024
Triglycerides, mmol/l	1.78 ± 1.30	1.75 ± 1.20	1.70 ± 1.09	0.290
LDL, mmol/l	2.68 ± 0.72	2.75 ± 0.80	2.67 ± 0.75	0.067
HDL, mmol/l	1.19 ± 0.33	1.20 ± 0.32	1.20 ± 0.31	0.723
TC, mmol/l	4.65 ± 1.01	4. 71 ± 1.04	4.61 ± 1.00	0.094
HBA1C, n (%)	6.34 ± 1.91	5.89 ± 1.87	5.87 ± 1.79	0.002
Hemoglobin, g/L	136.34 ± 16.96	137.84 ± 16.70	137.82 ± 16.68	0.091
Platelet, n/mm^3^	230.51 ± 62.02	226.03 ± 62.09	226.23 ± 64.24	0.223
STB, *μ*mol/l	6.73 ± 2.00	12.64 ± 1.61	17.76 ± 1.41	<.001
SDB, *μ*mol/l	3.52 ± 1.56	4.15 ± 1.18	4.53 ± 1.61	<.001
AST, u/L	28 (19, 35)	29 (19, 37)	29 (21, 37)	0.052
ALT, u/L	30 (19, 40)	32 (21, 43)	32 (20, 43)	0.007
*Previous medications*				
ACEI/ARB, n (%)	296 (32.22%)	286 (30.25%)	277 (30.01%)	0.633
*β*-blocker, n (%)	249 (26.98%)	233 (25.22%)	241 (26.11%)	0.690
Aspirin, n (%)	283 (30.66%)	432 (46.75%)	417 (45.18%)	<.001
Statins, n (%)	322 (34.89%)	453 (49.03%)	435 (47.13%)	<.001

LVEF: left ventricular ejection fraction; LDL: low-density lipoprotein; HDL: high-density lipoprotein; TC: total cholesterol; STB: serum total bilirubin; SDP: serum direct bilirubin; AST: aspartate aminotransferase; ALT: alanine aminotransferase; ACEI: angiotensin-converting enzyme inhibitor; and ARB: angiotensin II receptor blockade.

**Table 2:** The COX proportional hazards model analyses predictors for MACE within 30 days and 1 year.

Variables	30 days		1 year	
	HR (95% CI)	P-value	HR (95% CI)	P-value
STB group				
Group C	-	-	-	-
Group B	0.956(0.582-1.570)	0.859	1.139(0.746-1.740)	0.546
Group A	1.046(0.625-1.750)	0.865	2.002(1.342-2.986)	<.001
Troponin	1.079(1.050-1.109)	<.001	1.071(1.047-1.095)	<.001
LVEF	0.966(0.949-0.983)	<.001	0.947(0.935-0.960)	<.001
AST	0.998(0.996-0.999)	0.014	0.995(0.992-0.998)	0.002

LVEF: left ventricular ejection fraction; AST: aspartate aminotransferase.

**Table 3:** The COX proportional hazards model analyses predictors for cardiac death within 30 days and 1 year.

Variables	30 days		1 year	
	HR (95% CI)	P-value	HR (95% CI)	P-value
TBIL group				
Group C	-	-	-	-
Group B	0.355(0.084-1.505)	0.160	0.659(0.201-2.161)	0.491
Group A	2.148(0.724-6.372)	0.168	3.403(1.319-8.785)	0.011
Troponin	1.143(1.074-1.216)	<.001	1.126(1.070-1.185)	<.001
LVEF	0.932(0.893-0.973)	0.002	0.919(0.887-0.953)	<.001
AST	0.969(0.940-1.000)	0.049	0.982(0.965-0.999)	0.042
Hypertension	0.255(0.077-0.846)	0.026	-	-

LVEF: left ventricular ejection fraction; AST: aspartate aminotransferase.

**Table 4:** STB of patients that have each of other diseases.

Disease	STB (mmol/L)		Z	P
	None	With		
Hypertension	12.8 (8.9-16.7)	12.5 (8.1-16.4)	-1.634	0.102
Hyperlipidemia	12.6 (8.5-16.6)	12.6 (8.3-16.3)	-1.226	0.220
CAD	12.5 (8.9-16.3)	12.6 (8.3-16.5)	-0.833	0.405
Diabetes	12.7 (8.6-16.6)	12.2 (7.8-16.3)	-2.200	0.028
CED	12.6 (8.3-16.4)	12.8 (8.9-16.8)	-1.511	0.131
RI	12.6 (8.9-16.4)	12.6 (8.4-16.6)	-1.531	0.130

CAD: cardiovascular disease; CED: cerebrovascular disease; RI: renal insufficiency; none: patients without the disease; and with: patients with the disease.

## Data Availability

No data were used to support this study.
